# Pedicled buccal flaps as a backup procedure for intraoral reconstruction

**DOI:** 10.1007/s10006-022-01040-7

**Published:** 2022-01-24

**Authors:** Gesche Frohwitter, Marco R. Kesting, Andrea Rau, Manuel Weber, Christoph Baran, Christopher-Philipp Nobis, Tjark-Ole Buentemeyer, Raimund Preidl, Rainer Lutz

**Affiliations:** 1 Department for Oral and Maxillofacial Surgery, University Hospital Erlangen, Glueckstrasse 11, 91054 Erlangen, Germany; 2 Department for Oral and Maxillofacial Surgery, University Hospital Greifswald, Greifswald, Germany

**Keywords:** Pedicle flaps, Reconstructive surgery, Backup procedures, Cleft surgery, Oral surgery

## Abstract

**Background:**

Intraoral soft tissue deficiency and impaired wound beds are common problems after cleft and tumour surgery or after dental trauma. Frequently, limited defects are overtreated with extensive microvascular reconstruction procedures, but pedicled flaps remain useful, as they are simple to harvest, and they provide a reliable outcome. The buccal flap, first described in the 1970s, has been used for palatine lengthening in cleft patients over decades. In the following, we present an expanded indication in cases of palatal fistula, complex vestibulum, exposed bone in orthognathic surgery, and osteoradionecrosis.

**Methods:**

We conducted a retrospective chart review and report on all buccal flaps harvested in our department within the last 3 years with a follow-up period of at least half a year after flap surgery. Patients of all age groups and treatment indications in which a buccal flap was used were implicated in the evaluation.

**Results:**

Sixteen buccal flaps were performed in 10 patients. The median age at the time of surgery was 42 years, reaching from 12 up to 66 years. Fourteen buccal flaps were used for upper jaw or palatal coverage; two buccal flaps were used in the mandible. In terms of complications (four flaps; 25%), there were two partial flap failures, one wound dehiscence and one wound dehiscence. There were no failures of the remaining mucosal flap islands after pedicle dissection.

**Conclusion:**

The buccal flap is a reliable and straightforward approach to challenging intraoral wound beds with soft tissue deficiency. We thoroughly discuss the additional indications for buccal flap surgery, describe the harvest technique, and provide strategies to prevent intra- and postoperative complications.

## Introduction

Deficiency of intraoral soft tissue is often the result of ablative tumour surgery, trauma, or cleft formation. Considering the reconstructive ladder, there are two relevant strategies for circumscribed and medium-sized defects of the oral cavity: microvascular flaps or local (pedicled) flaps. The popularity of microvascular flaps due to their high success rates and flexible extend of defect coverage led to the development of the so-called pedicled (mini) perforator flaps during the last decade [1]. The advantage of minimal morbidity at the donor site of the perforator flap is offset by the non-mucosal lining for intraoral substitution as well as the short and small-diameter vascular pedicle, resulting in higher complication rates compared to conventional microvascular flaps [2, 3]. Furthermore, limited intraoral defects such as palatal fistulas in cleft surgery, mucosal shortage in complex dental implant procedures, or coverage of alveolar crest wounds in osteo (radio-) necrosis are not suitable for extensive microvascular reconstruction. As small intraoral defects may have a severe impact on the patients’ quality of life, such as fluid leakage through the nose in oronasal fistulas, thoughtful treatment planning is key to success as there is most frequently no easy approach to a localised defect in a scarred or irradiated wound bed so that also a small defect may be a challenge to treat. Standard procedures include (myo-) mucosal flaps like Axhausen’s cheek transposition flap or Rehrmann’s trapezoid mucoperiosteal buccal flap [4, 5]. However, they are restricted to defined anatomic locations such as oroantral fistula closure and may not be extended to palatal defects. Furthermore, in cases of secondary or tertiary surgery, the local tissue can be extensively impaired by scarring, malperfusion, and contraction, remaining impossible for standard local flap procedures. This led to the development of innovative (myo-) mucosal flaps in the late 1980s and early 1990s, which allow a wider range of movement to cover intraoral defects and are even successful in preoperated tissue [6–8]. Nevertheless, especially for the facial artery myomucosal flap, which resembles a nasolabial flap without a full-thickness skin coverage, donor site morbidity such as scar-induced trismus, aesthetic deficiencies due to buccal hollowing, and the severe complication of parotid duct injury make this flap questionable for small intraoral defects in young patients. Even though a random-pattern flap such as the bilateral buccal flap described by Mann et al. in 1997 has a less secure blood supply than an axial-pattern myomucosal flap, the technique has proven reliability especially in cleft patients, where compared to many tumour patients, the lack of radiation-induced fibrosis and scarring of the oral mucosa is an important advantage. They described the technique of bilateral buccal flaps for primary closure of wide palatal clefts and as a backup procedure for palatal lengthening in cases of cleft associated velopharyngeal dysfunction [9, 10]. The flap may be harvested as an either anteriorly or posteriorly pedicled random pattern myomucosal flap of the buccal soft tissue under sparing of the parotid duct. Depending on the local conditions it may have a length of up to 6 cm with a width of 2 cm, which allows a rotation just over the median sagittal plane and if harvested bilaterally safely covers the same.

Besides the primarily described indication of palatal lengthening, we successfully applied the buccal flap in recalcitrant maxillary and mandibular defects in cases of palatal fistula, complex vestibulum, and recurrent exposed bone in orthognathic surgery and osteoradionecrosis. To the best of our knowledge, the vast surgical options provided by this easy to harvest and reliable local flap are not discussed in the current literature. In the following, we report our own experiences with the pedicle flap as a backup procedure in heavily pretreated small defect coverage and outline a secure harvesting technique depending on the defect location.

## Methods

### Patients

Between October 2017 and September 2020, over 250 patients with small to medium-sized intraoral defects were treated by the use of microvascular and pedicled flaps in our institution. A retrospective chart review of the first authors’ experience (MK, GF) using the buccal flap as a backup procedure after (failed) surgical pretreatment was carried out. These included the demographic patient data, the initial (congenital) deformity or illness, previous therapies, and current chief complaints concerning the underlying defect. Additionally, records were checked on the buccal flaps’ pedicle location and postsurgical complications. The time of pedicle dissection was measured from the date of surgery to the date of surgical pedicle cut. The initially defined exclusion criteria, which was non-attended follow-up examinations at least 6 months after successful flap healing at the end of treatment, did not take effect, as all patients kept the appointments. Flap success was defined as the complete closure of the underlying defect with no further need for local revision. The review was performed in accordance with the Declaration of Helsinki and the approval by the local ethics committee (Medical Faculty of University Hospital of Erlangen, registration number 341_20Bc). Statistical analysis was performed using IBM SPSS Statistics Version 24 (Released 2016. IBM SPSS Statistics for Windows. Armonk, NY: IBM Corp.).

### Surgical technique

All patients underwent surgery in general anaesthesia with nasal intubation. If possible, the nasotracheal tube was placed in the nostril of the unaffected defect side. The wound margins of the defects were prepared by de-epithelialisation, sharp bone edges were removed, and the wound bed was rinsed with polyhexanide 0.04% (SERASEPT, SERAG-WIESSNER, Naila, Germany).

**Fig. 1:**
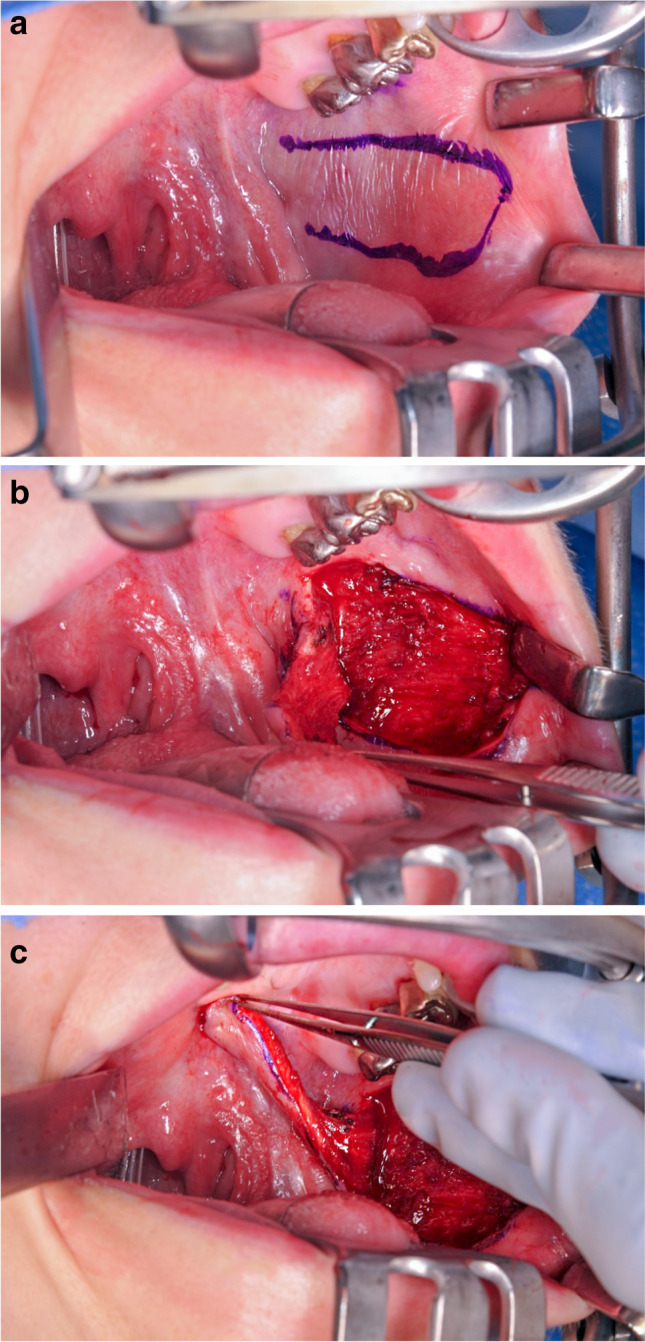
Workflow of a buccal flap harvest in three steps. **a** Marking of the incision lines; **b** buccal flap; harvest; **c** mobilisation of the buccal flap to the recipient site

For flap preparation, the parotid duct was marked, and the buccal flap size inferior to the parotid duct was outlined (Fig. 1). In cases of a posterior pedicle, the flap may not exceed the retromolar trigone. In anterior pedicled flaps, the base of the pedicle should respect a distance to the oral commissure of 1 cm to avoid a visible torsion of the lip and the corner of the mouth. The flap harvest was performed from distal to proximal by incision of the mucosa and a split dissection of the buccinator muscle with the leave of a thin muscle layer on the buccal fat (Fig. 1). Depending on the defect size and the individual anatomical circumstances, the pedicle may have a length of up to 6 cm with a diameter of 2 cm and a thickness of 0.5 cm (Fig. 1). After raising, the flap was rotated to the defect site, and bipolar cautery for haemostasis was performed. The donor site was closed primarily with resorbable sutures (SERAFIT 3–0, SERAG-WIESSNER, Naila, Germany) from distal to proximal saving a distance of 5 mm to the pedicle to prevent squeezing and malnutrition. The flaps were sutured to the defect site with resorbable sutures of either 3–0 or 4–0 USP-size (SERAFIT, SERAG-WIESSNER, Naila, Germany). All patients received nasogastric feeding tubes for 5 days and afterwards were instructed to keep a soft food diet until the cut of the pedicle. Furthermore, all patients were advised about careful oral hygiene by mouth rinse and brushing. Patients received a single-shot antibiosis during surgery or if considered necessary, for up to 10 days (ampicillin and clavulanic acid, for patients with penicillin allergy: clindamycin). Analgesics were prescribed according to the WHO scheme and included nonsteroidal anti-inflammatory drugs and metamizole.

All patients were integrated into clinical recalls. The pedicle was left in place until complete local wound closure and flap autonomy was achieved. Flap autonomy was assessed based on clinical impression and a colour change when the pedicle was gently squeezed with forceps. In a second procedure, the pedicle was transected under general or local anaesthesia in dependence on the further treatments necessary. At the reconstructive site, the tissue was sutured to the local mucosa (resorbable sutures 3–0 or 4–0 USP-size, SERAFIT, SERAG-WIESSNER, Naila, Germany), the tissue excess at the donor site was usually not resutured but resected and cauterised, as it frequently results as a trigger for cheek bites.

## Results

A total of 16 buccal flaps, 6 bilaterally and 4 unilaterally, in 10 patients (5 females, 5 males) were harvested (Table 1). The median age at the time of surgery was 42 years (minimum 12 years, maximum 66 years, mean 48 years). One patient died 83 days after flap surgery due to distant metastatic tumour progress. All patients (*n* = 10) who received buccal flaps were extensively surgically pretreated within the oral cavity (minimum 5 times, maximum 35 times). Five patients initially suffered from malign oral tumour disease or precancerous lesion, three patients received multiple cleft surgeries, one patient experienced complex dental trauma, and one patient suffered from the exposed bone after orthognathic surgery.

**Table 1:** Clinical data of all patients treated with a buccal flap

Patient/pedicle number (sex)	Age	Diagnosis	Pedicle location	Flap width (cm)	Flap length (cm)	Defect location	Pedicle cut (days)	Wound healing disorder	Surgical revision	Pedicle cut	Successful wound closure
1/1 (f)	58	Palatal fistula after cleft repair	Anterior	2.0	5.5	Palate	36	No	No	GA	No
2/1 (f)	20	Palatal fistula after cleft repair	Anterior	1.0	3.5	Palate	133	No	No	GA	Yes
2/2 (f)	20	Palatal fistula after cleft repair	Anterior	1.5	3.5	Palate	133	No	No	GA	Yes
3/1 (m)	12	Palatal fistula after cleft repair	Posterior	2.0	4.0	Palate	23	No	No	GA	Yes
4/1 (m)	66	Vestibuloplasty after oral cancer↯	Posterior	2.0	6.0	Mandible vestibulum	35	No	No	GA	Yes
5/1 (m)	63	Vestibuloplasty after oral cancer↯	Anterior	2.0	4.5	Maxilla anterior alveolar ridge	47	No	No	LA	Yes
5/2 (m)	63	Vestibuloplasty after oral cancer↯	Anterior	2.0	5.0	Maxilla anterior alveolar ridge	47	No	No	LA	Yes
6/1 (f)	52	Palatal fistula after tumour resection	Posterior	2.0	5.5	Palate	106	Refixation of the pedicle	Yes	GA	Yes
6/2 (f)	52	Palatal fistula after tumour resection	Posterior	2.0	5.5	Palate	106	No	No	GA	Yes
7/1 (f)	47	Palatal fistula after tumour resection	Posterior	1.5	5.5	Palate	No	Partial flap failure, no soft food diet	Yes	No	Yes
7/2 (f)	48	Backup flap after partial buccal flap failure	Posterior	1.8	5.0	Palate	No	Clinical irrelevant palatal fistula	No	No	No
8/1 (m)	59	Exposed bone after osteoradionecrosis	Anterior	1.5	3.5	Mandible crest	No	No	No	No	Yes
9/1 (m)	36	Autologous bone graft coverage after TPD	Anterior	1.5	5.0	Maxilla, anterior alveolar ridge	30	Partial flap failure, smoker	Yes	GA	Yes
9/2 (m)	36	Backup flap after partial buccal flap failure	Anterior	1.5	5.0	Maxilla, anterior alveolar ridge	26	No	No	GA	Yes
10/1 (f)	19	Autologous bone graft coverage after dental trauma	Anterior	1.5	4.0	Maxilla, anterior alveolar ridge	36	No	No	LA	Yes
10/2 (f)	19	Autologous bone graft coverage after dental trauma	Anterior	1.5	4.0	Maxilla, anterior alveolar ridge	No	No	No	No	Yes

Abbreviations: *TPD*, transpalatal distraction; *GA*, general anaesthesia; *LA*, local anaesthesia; ↯, radiation therapy

**Fig. 2:**
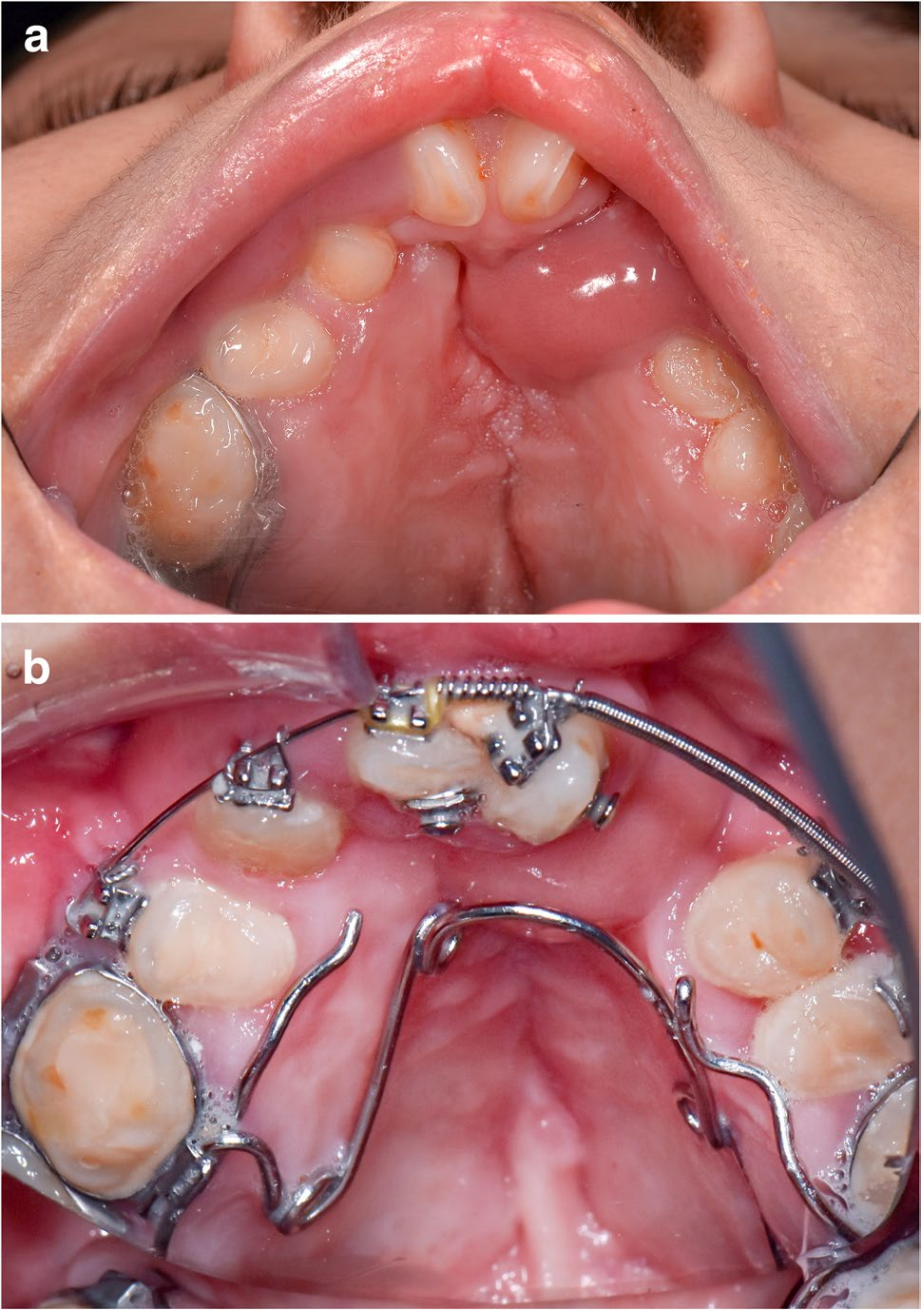
A 14-year-old patient with a bilateral palatal fistula after bilateral cleft repair after fistula closure. **a** Healed bilateral buccal flap in a case of successful palatal fistula coverage in a bilateral cleft lip and palate; **b** healed bilateral buccal flap with an inserted orthodontic multiband appliance

**Fig. 3:**
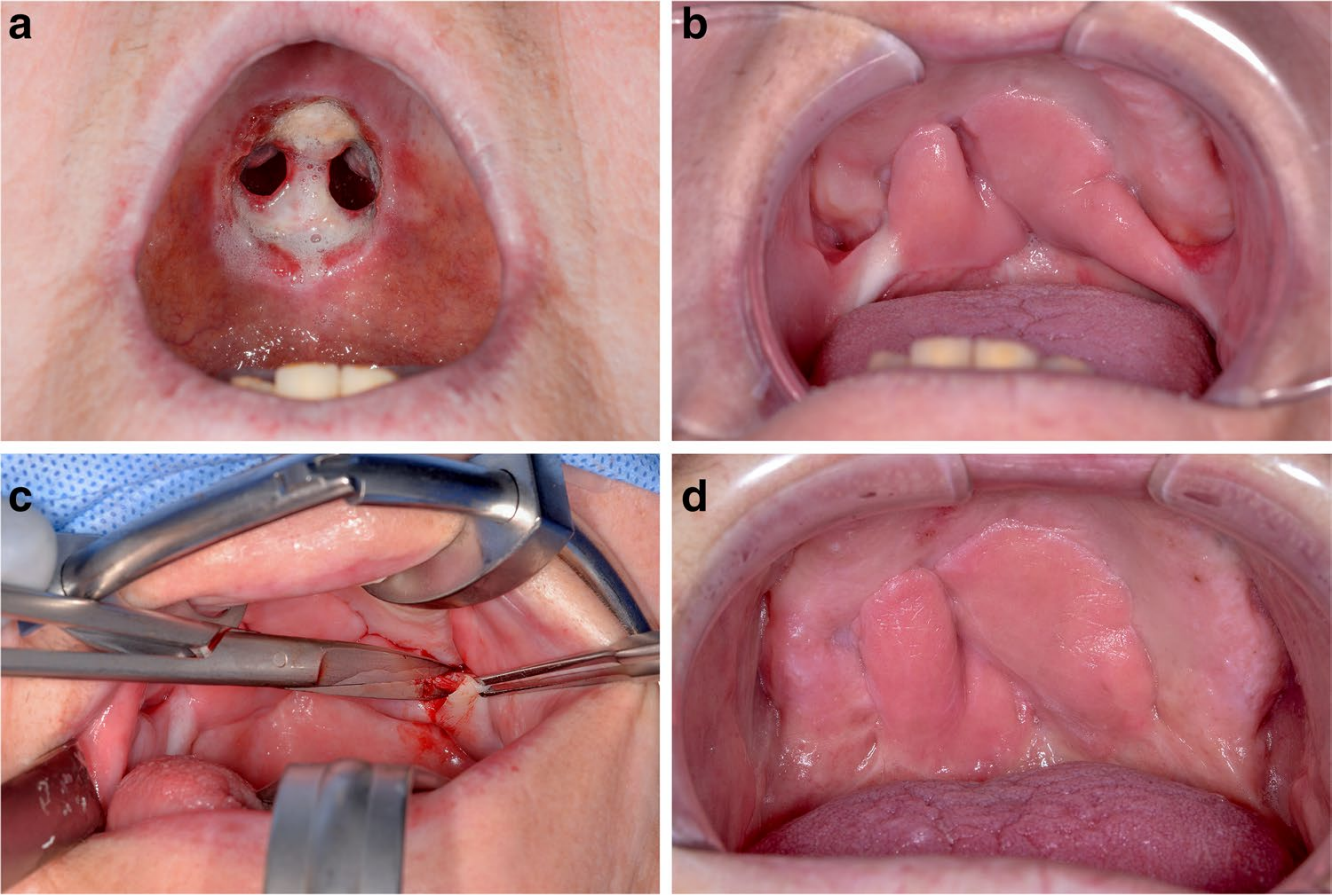
A 53-year-old patient with a palatal fistula after tumour resection. **a** Hard palate wound ground after tumour resection; **b** pedicled buccal flap adapted to the recipient site of the hard palate; **c** pedicle dissection; **d** hard palate with healed bilateral buccal flap

The indication for buccal flap surgery (*n* = 16) were four palatal fistula closures after cleft surgery (25%; no flap failure wound dehiscence; Fig. 2), three palatal fistula closures after tumour resection and wound dehiscence of a microvascular transplant (18.75%; no flap failures, one persisting palatal fistula; Fig. 3), three vestibuloplasties after tumour surgery (18.75%; no flap failures), three mucosal coverages after bone autogenous bone augmentation (18.75%; one partial flap failure due to malperfusion), one mucosal coverage after osteoradionecrosis resection (6.25%; no flap failure), and two surgical wound revisions after previous partial buccal flap failure (12.5%; one flap failure due to wound dehiscence). Fourteen buccal flaps (87.5%) were used for the upper jaw or palatal coverage, and two buccal flaps (12.5%) were used in the mandible. Ten flaps (62.5%) were anterior pedicled, and 6 flaps (37.5%) were posterior pedicled. The flap sizes had a mean width of 1.7 cm and a mean length of 4.7 cm (width: minimum 1.0 cm, maximum 2 cm, median 1650 cm; length: minimum 3.5 cm, maximum 6 cm, median 5.0 cm). In terms of complications (four flaps; 25%), there were two partial flap failures (12.5%) that required the raising of a second buccal flap from the contralateral side, one wound dehiscence (6.25%) that required a surgical intervention to refixate the mucosal island, one wound dehiscence (6.25%) with a clinical irrelevant palatal fistula that was left untreated, and no total flap losses. The complications were not radiation associated but resulted from either a lack of compliance such as smoking and no soft food diet or from a difficult local tissue situation due to multiple previous surgeries. In cases of pedicle interference with the occlusion, a removable occlusal splint was inserted to prevent squeezing.

The pedicle was cut in 12 cases (*n* = 16, 75%); in 4 cases (25%), the pedicle remained as it completely adapted to the former wound bed. The mean time form flap raises to cutting the pedicle was 63.17 days (median 41.5 days, minimum 23 days, maximum 133 days). In the case where the pedicle was cut 133 days after the initial flap raise, the patient received further orthodontic and medical treatment due to physical disabilities (CHARGE syndrome), which postponed the maxillofacial surgery. There were no failures of the remaining mucosal flap islands after the cut of the pedicles. Three pedicles (23.07%) were cut in local anaesthesia, and ten pedicles (76.92%) were cut under general anaesthesia as further surgical interventions were required. A secondary flap debulking after the cut of the pedicle did not become necessary in any patient. In two cases (12.5%), the palatal fistula of a tumour patient and a cleft patient could not be closed sufficiently by two buccal flaps; all other flaps showed the desired results and long-term stability. None of the patients experienced any donor site morbidity such as intraoral deformities causing cheek bites, speech difficulties, chronic pain, or reduced mouth opening.

Table 1 offers an overview of all buccal flap procedures evaluated in this article.

## Discussion

The current study indicates the myomucosal random patterned buccal flap as an excellent backup procedure for defect closure of small recalcitrant wounds in the maxilla, the palate, and the mandible, which will be discussed in the following.

Introduced in the mid-1970s, the buccal flap has experienced many modifications [11, 12]. It can be harvested as an axial pattern flap including the facial artery or the buccal artery as the main vessel as well as a random pattern flap. Furthermore, its thickness may reach from thin myomucosal flaps up to pedicled buccal fat pad integration [8, 13, 14]. This variety in harvesting techniques and flap thickness offers a broad field of application in tension-free, watertight, and localised defect closure. Apart from its traditional indication for primary closure of wide palatal clefts or as a backup procedure for palatal lengthening in velopharyngeal dysfunction, many case reports on the topic have been published [15, 16]. In 1989, Bozola et al. reported a cadaver and clinical study on the axial patterned buccinator musculomucosal flap, which had been successfully applied not only in cleft surgery but also in tumour surgery and osteomyelitis [14]. However, up to our experience, the technique of a thick axial-patterned flap in the oral cavity results in many adverse side effects such as slurred speech, nutritional problems, or aesthetical deficiencies of the cheek volume that make secondary flap thinning indispensable. Besides, the accidental injury of the parotid duct is more frequent. Hence, we posed the question of whether a thin and less invasive random patterned flap may serve as an adequate alternative for a multitude of localised wounds in the oral cavity.

We used the buccal flap in ten patients who overall received 16 flaps and had undergone a multitude of surgeries in advance (minimum 5 times, maximum 35 times). The surgical indications were the closure of palatal fistulas after tumour resection and cleft surgery, pre-implantological soft-tissue improvement by vestibuloplasties, mucosal covering of autogenous bone transplants, and defect closure after the resection of osteoradionecroses. Most flaps were harvested for defect closure in the maxilla and the palate, which is due to the fact that the lower jaw provides a wider range of movable tissue not only from the vestibulum but also from the floor of the mouth, that lacks the rigid keratinized mucosa of the hard palate and offers a broader range of flap alternatives such as the mylohyoid muscle flap [17]. The surgeries were uneventful; all flaps were harvested without complications such as malperfusion, injury of the pedicle, or the parotid duct and suited the initial treatment indication. In two cases, the palatal fistulae could not be closed completely but were transposed from clinically apparent (nasal food and fluid regurgitation) to clinically silent (no food and fluid regurgitation). Both patients were heavily pretreated with up to 35 surgical attempts in different hospitals and private practices to close the fistulae. However, when considering a thin random-patterned buccal flap as an option for intraoral soft tissue reconstruction, certain aspects need to be borne in mind.

Firstly, the defect location determines the origin of the pedicle. Anterior-pedicled flaps may be indicated in tissue shortage of the anterior half of the oral cavity up to the first premolar and cover the entire vestibulum and hard palate of the harvested flap site, whereas posterior pedicled flaps mostly serve as coverage for the dorsal half of the oral cavity including the soft palate. In both cases, distal flap perfusion seems to be limited when the myomucosal flap extends a length-to-base relation of over 3:1. To preserve the aesthetic unit of the lips and prevent extraoral deformities due to tension or distortion, a gap of 1 cm to the angle of the mouth should be kept in anteriorly pedicled flaps. In posteriorly pedicled flaps, the retromolar trigone should not be incised to prevent bleeding and secure sufficient flap perfusion. Independently of the pedicle location, a flap mobilisation over the median sagittal plane is not recommended as pulling and stressing the pedicle results in immediate malperfusion followed by dehiscence or flap loss. Due to masticatory movements and speech, which often result in the tension of the pedicle, buccal flaps for tissue coverage in the lower jaw are more sensitive for wound healing disorders than defect closures in the upper jaw. However, in both cases, as soon as the flap crosses, the alveolar crest or the teeth the need of a spacer in the form of an occlusal splint needs to be considered to avoid biting and injuring the pedicle. During the mixed dentition period, tooth gaps may provide a natural corridor, which prevents pedicle squeezing.

We found the random pattern nutrition as no disadvantage in secure flap perfusion and performed a vessel independent harvest even in patients with heavily pretreated and perfusion-compromised tissue. Especially in hostile necks where the lack of facial arteries or the damage of both lingual arteries may lead to necrosis of tongue flaps or reduced security in nasolabial flaps, the buccal flap is a reliable alternative [18, 19]. It usually offers a larger and more versatile amount of movable tissue in cases of extensively impaired intraoral wound beds, commonly seen after secondary or tertiary closure in palatal clefts, oroantral fistulae, osteo(-radio) necrosis, and as a lack of attached gingiva or vestibulum depth after ablative tumour surgery compared to classical local flaps such as Rehrmann’s trapezoid flap or Axhausen’s cheek flap. However, a buccal flap harvest in a patient with radiation-induced trismus and/or heavy oral respiration and xerostomia should be rethought, as these factors are highly prone to cause flap failure.

Additional, meticulous wound care is needed to promote uneventful healing. Therefore, we highly recommend nasogastric tube feeding for at least 5 days, regular mouth rinsing with Chlorhexamed 0.2%, and careful toothbrushing. When the nasogastric tube is removed, a soft diet should be kept until the cut of the pedicle. Current literature provides inconsistent data concerning the time of transplant autonomation, reaching from 2 to 6 weeks [20, 21]. Depending on the local wound situation, our patient collective showed a very wide time range of 63.17 days (median 41.5 days, minimum 23 days, maximum 133 days) for pedicle incision. Commonly, we test the autonomic flap perfusion by clinical evaluation. If the defect-covering flap part does not significantly change in colour whilst being squeezed by forceps, we consider the pedicle as dispensable. The maximum time of division (133 days) was due to a prolonged orthodontic treatment to prepare the upper jaw for cleft osteoplasty with an autogenous bone transplant and exceeded the clinical appearance from which point on a cut of the pedicle would have been uneventful. Furthermore, the patient suffers from a CHARGE syndrome that demanded medical intervention during which no maxillofacial treatment was possible. In four cases, a pedicle removal was not performed as the lower surface of the flap was completely sutured to the wound bed and aligned with the local mucosa. Nevertheless, the two-stage removal of the pedicle may be seen as the main disadvantage in buccal flap treatment. The intraoral wound situation appears inconvenient and presurgical patient education and careful patient selection are needed to ensure the best conditions for uneventful healing. As all patients in our collective showed a long history of surgical and adjuvant pretreatment, the buccal flap served as a final backup for local would management before referring to microvascular tissue replacement. This aspect needs a high patient motivation but justifies the inconvenient time of intraoral pedicle carriage and the temporary restrictions to nutrition, speech, and oral hygiene. In our experience, the pedicled buccal flap represents a reliable and versatile alternative in selected cases of the impaired wound bed and intraoral soft tissue scarcity in demanding local situations for oral and maxillofacial surgeons.

## Conclusion

The article introduces a new field of application for the myomucosal random-patterned buccal flap in heavily pretreated intraoral soft-tissue defect coverage. Considering the pieces of advice in patient selection, flap raise, and postsurgical aftercare, it provides a secure and technically straightforward alternative in a highly demanding niche in intraoral maxillofacial backup reconstruction.
